# Conjoint analysis of social inequality indicators in diet and physical activity app (non-)users: Representative online surveys in Austria, Germany and Italy

**DOI:** 10.1177/20552076261429621

**Published:** 2026-03-26

**Authors:** Laura M König, Lucia Volpi, Theresa JS Koch

**Affiliations:** Faculty of Psychology, 31289 University of Vienna, Vienna, Austria

**Keywords:** Digital health, mHealth, socio-economic factors, health promotion

## Abstract

**Background:**

Mobile interventions for health promotion (mHealth) are promising behaviour change tools. Yet they are infrequently used, and research suggests that use may be unevenly distributed in the population, potentially widening existing health inequalities.

**Objective:**

This study tested for individual and joint associations between socio-demographic characteristics and nutrition and physical activity app use.

**Methodology:**

Nationally representative samples for Austria, Germany and Italy were recruited with *N* = 1974 participants in total. In an online survey, participants reported on nutrition and physical activity app use as well as a range of relevant socio-demographic characteristics associated with social inequality according to PROGRESS-Plus (age, gender, education, income, employment status, rural vs. urban residency, Austrian/German/Italian citizenship, migration history, minority status, and sexual orientation).

**Results:**

Except for residency and migration status, all socio-demographic characteristics were associated with mHealth app (non-)use if analysed independently. A latent class analysis revealed four distinct classes of mHealth app (non-)users. ‘Young and diverse citizens’ (characterised by young age and lowest proportion of heterosexuals) and ‘economically strong employees’ (characterised by highest levels of education and income) were more likely to use mHealth apps compared to ‘established retirees’ (characterised by largest share of retired individuals) and ‘low-income workers’ (characterised by lowest levels of education and income).

**Conclusion:**

Age, education, income and employment are crucial inequality indicators for mHealth app use. These results confirm the existence of a digital health divide in Europe that urgently needs addressing to promote digital health for all.

**Trial registration:**

https://osf.io/s2wya (Austria and Germany), https://osf.io/tpu2m (Italy).

## Background

Digital health interventions use digital technology to educate about health and to promote health literacy and behaviour change. They allow to reach many people at relatively low cost, providing support in real-life and real-time when it is most needed. Indeed, digital health interventions are effective in promoting health literacy^1,2^ and in changing a range of health behaviours, including eating and physical activity^3^ in various populations and settings; this is also true for interventions that are delivered exclusively via mobile (mHealth) apps.^4^ Public health authorities, including the World Health Organization^5^ and institutions such as the European Union,^6–8^ advocate for widely implementing digital solutions in health education, promotion and care.

Despite the potential public health benefits of mHealth interventions, the use of mHealth apps is limited in many areas of the world. Use rates range from 21% in Japan to 70% in India.^9^ Apps addressing diet and physical activity are among the most popular^10,11^ and may provide an important lever to curb rising overweight rates. However, evidence is accumulating that mHealth app use and engagement are not equally distributed across the population. For example, diet and physical activity apps are more frequently used by women compared to men,^12–14^ by younger compared to older adults,^14–17^ and by individuals with higher compared to lower educational attainment.^18–20^ These findings point towards a digital health divide,^21^ which is rooted in limited access to and knowledge about mobile health tools.^22^ For example, older adults and individuals with lower educational attainment may not be as experienced in using digital tools and therefore may not consider using them for health promotion. This may further exacerbate existing health inequalities.

According to the Cochrane PROGRESS-Plus framework,^23^ a whole range of socio-demographic characteristics are associated with inequalities in healthcare, including digital health. Yet, most of these are only rarely studied in relation to diet and physical activity app uptake or not at all.^24–26^ Closing this evidence gap is important to be able to draw conclusions about potential additional factors contributing to the digital health divide that may need addressing. Furthermore, most available data on the digital health divide in relation to diet and physical activity apps stems from the US, and relatively little data is available for other regions of the world, including Europe,^26^ where overweight and obesity are still on the rise and thus require urgent attention.^27^ Finally, most available studies address a small number of social inequality indicators and investigate them separately, neglecting the fact that they might be interdependent. For example, education and income may affect each other, potentially leading to a vicious circle.^28^ Taking these potential interdependencies into account will provide valuable insights into which underlying factors of the digital health divide to address, to promote (digital) health for all.

To the best of our knowledge, the present research is the first comprehensive, multi-country study in Europe conjointly assessing a broad range of social inequality indicators with regards to diet and physical activity app (and wearable) use in adults. The study specifically focused primarily on apps and did not include other types of mHealth interventions, because most individuals in the studied countries own a smartphone and thus can use mHealth apps without necessarily having to purchase another device.^29^ The study specifically addressed the following research questions. First, we aimed to understand whether the use of nutrition and physical activity-related apps differed between socio-demographic groups derived from the PROGRESS-Plus framework of social inequality indicators, focusing on gender, age, education, income, employment, residency, migration status, discrimination experiences, and sexual orientation. Based on the available literature,^14,25^ it was hypothesised that women are more likely to use diet apps, and that current and former diet and physical activity app users are younger than non-users. No hypotheses were formulated for the other social inequality indicators due to inconsistent findings in the literature.^24,25^ Second, we aimed to investigate potential interdependencies between social inequality indicators in relation to diet and physical activity app use by conducting a conjoint analysis, that is, taking into account all inequality indicators simultaneously in a latent class analysis. This research question was exploratory. Data was collected in Austria, Germany and Italy to close the data gap for European countries, and representative samples based on age, gender and level of education were recruited to generate generalisable insights.

## Methods

For this analysis, we used data from two larger surveys on digital health and health behaviours conducted in Germany and Austria in spring 2024, and Italy in summer 2024. Although all three countries are high-income countries^30^ in central Europe with net migration,^31^ the Italian population has a somewhat lower socio-economic status as indicated by higher unemployment rates,^32^ lower income as indicated by GDP per capita,^33^ and lower rates of tertiary education,^34^ while at the same time reporting higher life expectancy^35^ and lower lifestyle risk factors (e.g. obesity^36,37^) than the Austrian and German populations.

The surveys were preregistered prior to data collection on the OSF.^38,39^ The data analysis plans for this study was preregistered before the data were collected.^40,41^ Materials, data and code are available from the OSF (Austria and Germany: https://osf.io/swupm/; Italy: https://osf.io/xqruz/).

The study was carried out in accordance with the Declaration of Helsinki. Ethical approval was obtained from the University of Vienna ethics committee (approval numbers 01141 and 01195).

### Sample

We aimed to recruit 1000 participants from Germany, 500 participants from Austria and 500 participants from Italy via a panel provider. No formal power calculation was conducted a priori; instead, the sample size was determined based on available funding. With a sample size of about *N* = 2000, we are able to detect effects of Cohen's *f* = 0.07 (one-way between-subjects ANOVA with five groups, two-sided) or *w* = 0.08 (χ^2^ test with *df* = 4; all α = .05) at 80% power (cf. G*Power 3.1, Faul et al.^42^).

Samples were stratified by age, gender and level of education based on the respective national statistics (see Table 1). For Germany and Austria, adults aged 18 years and older were eligible; for Italy, the sample was restricted to 18–50 years due to its primary focus on studying pre-menopausal women. Participants were only recruited from these three countries. No further eligibility criteria were applied.

**Table 1.** Comparison of the subsamples’ age, gender and level of education to German and Austrian national statistics.

Sample characteristic	Full sample (*N* = 1974)	German subsample (*N* = 975)	German national statistics	Austrian subsample (*N* = 487)	Austrian national statistics	Italian subsample (*N* = 512)	Italian national statistics
Age *M* (*SD*)	45.06 (15.97)	48.62 (16.67)	46.5	47.51 (16.62)	43.3	35.96 (8.95)	46.6
Gender: Women	49.6%	50.1%	50.71%	49.9%	50.7%	48.4%	51.1%
Gender: Men	50.1%	49.5%	49.29%	50.1%	49.3%	51.0%	48.9%
Gender: Other	0.4%	0.4%				0.6%	
Tertiary education	26.7%	27.1%	32.5%	31.0%	35.6%	22.1%	20.8%

*Note:* The German national statistics regarding age and gender stem from Statistisches Bundesamt,^43,44^ and regarding tertiary education from OECD.^45^ For Austria, these data stem from Statistik Austria^46^ (age), Bundeskanzleramt^47^ (gender), and OECD^45^ (tertiary education). For Italy, these data stems from Istituto Nazionale Di Statistica.^48^

### Design and procedure

In each country, we conducted a cross-sectional survey in the official language (German and Italian, respectively). Participants provided informed consent by ticking a box on the first page of the online survey. They first provided socio-demographic (cf. social inequality indicators) and anthropometric information. They then completed a set of questionnaires on psychological characteristics; ownership of digital devices; use of smartphone apps to track diet, physical activity and the menstrual cycle (for women); menstrual symptoms (for biologically female participants) and attitudes towards menstrual leave. In the German and Austrian sample, participants additionally indicated their intentions to lose weight and change their diet; comfort foods; the type, frequency and duration of physical exercise; two vignettes presenting scenarios of the participants playing sports with friends of different skill levels (in random order), and attitudes towards and use of exergames; and use of and attitudes towards leaderboards in fitness apps. They were then debriefed and redirected to the panel provider for payment. The present analysis focuses on social inequality indicators, digital device ownership, and use of diet and physical activity apps. The remaining data is presented elsewhere.^49–51^

### Materials and measures

#### Smartphone and tablet ownership and use of diet and physical activity apps

Smartphone and tablet ownership was assessed (both yes/no) as a prerequisite of being able to use nutrition and physical activity apps. All participants who indicated that they owned a smartphone or tablet were then asked about their diet and physical activity app use using a stage model with five stages^14^ based on the precaution adoption process model.^52,53^ The questionnaire has been developed in another German sample^14^ and includes the following items: (1) ‘I have never thought about using an app for that [nutrition/physical activity]’ (unengaged non-users); (2) ‘I have thought about using an app for that [nutrition/physical activity], but so far I did not do it’ (non-users decided to act); (3) ‘I have thought about using an app for that [nutrition/physical activity], but it is not necessary for me to do it’ (non-users decided not to act); (4) ‘I am currently using an app for that [nutrition/physical activity] and intend to continue to use it’ (users); and (5) ‘I have used an app for that [nutrition/physical activity], but I do not use it anymore’ (disengaged non-users). Participants were asked to select the response that described them best, and they responded for diet and physical activity apps separately. For physical activity apps, apps that are used to access physical activity data from wearables were explicitly included.

#### Social inequality indicators assessed via socio-demographic information

We assessed a range of social inequality indicators, including age, gender, education, income, employment status, rural versus urban residency, Austrian/German/Italian citizenship, migration history, and sexual orientation, using items adapted from the literature, including other large-scale surveys conducted in Germany as well as item recommendations for studies on social inequality and diversity.^54,55^ Since data on race and ethnicity cannot be assessed in many European countries for historic reasons, we assessed self-identification with an ethnic minority status via discrimination experiences, as recommended by Stadler et al.^54^ Years of education were calculated as described in the respective data analysis plans.^40,41^ Monthly net household income was divided into quartiles as preregistered to ensure approximately equal group sizes. Migration status was determined based on the participants’ and their parents’ migration history: they were classified as migrants if they themselves or at least one of their parents migrated to Austria (if they currently lived in Austria), Germany (if they currently lived in Germany) or Italy (if they currently lived in Italy). Since the number of participants indicating sexual orientations other than being heterosexual was very small, which would have led to vastly imbalanced groups (see Table 2), sexual orientation was recoded into a binary variable, coding for whether individuals identify as heterosexual (1 = yes; 0 = no).

**Table 2.** Sample characteristics and demographics.

Sample characteristics	Total		Germany		Austria		Italy	
	*N*	%	*N*	%	*N*	%	*N*	%
Rural versus urban residency								
Large city	691	35	342	35.1	175	35.9	174	34
Medium-sized	384	19.5	241	24.7	28	5.7	115	22.5
Town	406	20.6	219	22.5	58	11.9	129	25.2
Village	493	25	173	17.7	226	46.4	94	18.4
Citizenship								
Yes	1909	96.7	950	97.4	458	94	501	97.9
No	65	3.3	25	2.6	29	6	11	2.1
Ethnic minority status								
Yes	148	7.5	77	7.9	39	8	32	6.3
No	1778	90.1	879	90.2	430	88.3	469	91.6
Prefer not to say	48	2.4	19	1.9	18	3.7	11	2.1
Migration status								
Yes	280	14.2	146	15	91	18	43	8.4
No	1694	85.8	829	85	396	81	469	91.6
Highest school leaving certificate (Italy)								
Elementary school							1	0.2
Secondary school leaving certificate							129	25.2
Graduation from vocational school							28	5.5
High school diploma							241	47.1
University degree (bachelor's)							48	9.4
University degree (master's)							48	9.4
Post-university qualification (PhD/doctorate)							17	3.3
Highest school leaving certificate (Germany/Austria)								
No school-leaving certificate			11	1.1	1	0.2		
Elementary school			152	15.6	65	13.3		
Secondary school leaving certificate			388	39.8	33	6.8		
Polytechnic secondary school			37	3.8	17	3.5		
Vocational technical school			92	9.4	225	46.2		
High school diploma (Abitur/Matura)			290	29.7	139	28.5		
Other			5	0.5	7	1.4		
Highest vocational qualification (Germany/Austria)								
No vocational training			170	17.4	81	16.6		
Apprenticeship			416	42.7	106	21.8		
Technical/vocational secondary school			92	9.4	149	30.6		
Degree from the University of Applied Science			96	9.8	41	8.4		
University degree			155	15.9	68	14		
PhD/doctorate			14	1.4	14	2.9		
Other			32	3.3	28	575		
Net household income								
< 150 €	33	1.7	13	1.3	9	1.8	11	2.1
150–300 €	17	0.9	6	0.6	5	1	6	1.2
300–500 €	30	1.5	13	1.3	7	1.4	10	2
500–1000 €	134	6.8	65	6.7	25	5.1	44	8.6
1000–1500 €	263	13.3	113	11.6	46	9.4	104	20.3
1500–2000 €	282	14.3	117	12	50	10.3	115	22.5
2000–2500 €	245	12.4	130	13.3	61	12.5	54	10.5
2500–3000 €	274	13.9	134	13.7	69	14.2	71	13.9
3000–5000 €	527	26.7	285	29.2	163	33.5	79	15.4
5000–10,000 €	150	7.6	92	9.4	45	9.2	13	2.5
> 10,000 €	17	0.9	6	0.6	7	1.4	4	0.8
Employment								
Full-time job	855	43.3	409	41.9	170	34.9	276	53.9
Part-time job	320	16.2	157	16.1	90	18.5	73	14.3
Job seeker	153	7.8	56	5.7	27	5.5	70	13.7
Student	152	7.7	63	6.5	35	7.2	54	10.5
Homemaker	88	4.5	33	3.4	18	3.7	37	7.2
Retired	406	20.6	257	26.4	147	30.2	2	0.4
Sexual orientation								
Heterosexual	1738	88	843	86.5	426	87.5	469	91.6
Homosexual	80	4.1	45	4.6	23	4.7	12	2.3
Bisexual	93	4.7	50	5.1	21	4.3	22	4.3
Pansexual	11	0.6	5	0.5	4	0.8	2	0.4
Asexual	17	0.9	9	0.9	6	1.2	2	0.4
Other	8	0.5	7	0.7	0	0	2	0.4
Prefer not to say	53	2.7	28	2.9	18	3.7	7	1.4

### Statistical analysis

Analyses were conducted in SPSS version 29.0.1 and MPlus version 7.4. As preregistered, we provide descriptive statistics for all socio-demographic variables and for the diet and physical activity app use stages. Since all questions were mandatory, there was no missing data due to missed responses. For both sexual orientation and discrimination experiences, which were used as a proxy for ethnic minority status, ‘no response’ was coded as missing; this resulted in 2.7% missings for sexual orientation and 2.4% missings for discrimination experiences.

#### Analyses testing for associations between mHealth (non-)use and social inequality indicators

To test relationships with the individual social inequality indicators, the following analyses were conducted separately for diet and physical activity app use stage, but combining the samples from all three countries. For continuous dependent variables (age and years of education), one-way between-subjects ANOVAs were conducted; in case that the homogeneity of variances assumption was violated, Welch's ANOVAs were conducted instead (deviating from the preregistration). Significant main effects were followed up with Bonferroni-corrected post-hoc tests. For categorical and ordinal dependent variables (gender, net household income, employment, rural versus urban residency, migration status, discrimination experiences and sexual orientation), chi-square tests of independent were conducted. For the analyses involving gender, due the small number of participants not identifying as a man or a woman, participants indicating the option ‘other’ were removed. For all analyses, significant results were followed up using pairwise Z-tests. Missings were deleted listwise. Diverging from the preregistration, we also explored country differences using a chi-square test and pairwise Z-tests. For all tests, the Type 1 error was set to 0.05.

#### Conjoint analysis

Finally, to explore the interplay of social inequality indicators, we conducted latent class analyses. This method allowed us to identify subgroups within the population (i.e. latent classes) that are characterized by an interplay of specific values in the respective social inequality indicators. The analysis included two main steps: (1) latent class identification, and (2) outcome analyses.

#### Data preparation for conjoint analyses

Given our data was from three countries, we planned to compute models with the pooled data (i.e. including data from all three countries) as well as country-level models (i.e. separate models for Germany, Austria and Italy each; cf. Kácha et al.^56^) and decide by means of invariance testing whether to proceed with one combined or two separate models. Diverging from this preregistered procedure, however, we directly tested the combined model since the country-level models hardly converged when increasing the number of classes (in spite of adapting other components, such as increasing the number of random starts or changing the estimator). We assume that these convergence issues arose due to high model complexity (i.e. many social inequality indicators included), which usually requires large sample sizes that likely only the pooled data set provided. We opted for only testing the combined model instead of reducing the number of social inequality indicators (which might have reduced model complexity) to match our aim to investigate the *interplay of social inequality indicators* (which was still possible in the overall model without dropping some of the indicators).

#### Latent class identification

Proceeding with the overall model (i.e. including data from all three countries), we then explored in the first step (i.e. latent class identification) how participants in our sample divided into different classes based on the social inequality indicators (i.e. gender, age, years of education, net household income, employment status, rural/urban residency, migration status, discrimination experiences and sexual orientation). The aim of this step was to identify distinct latent classes, in which members were relatively homogeneous regarding parameter values of the social inequality indicators, but heterogeneous when being compared to other latent classes within the overall population.

To uncover how many latent classes exist in the population based on the social inequality indicators, we successively tested models with a growing number of classes and compared fit indices. Thereby, we planned to increase the number of classes until the fit indices of the models aggravate or models do not converge anymore, which was the case after testing the model with six classes. For the fit indices, we followed recommendations^57,58^ and previous related research (e.g. Kácha et al.^56^), suggesting to rely on Akaike information criterion (AIC), Bayesian information criterion (BIC), sample-size-adjusted BIC (SSA-BIC), entropy, and on the Lo-Mendell-Rubin adjusted test (LMR test). For AIC, BIC, and SSA-BIC, smaller values are interpreted in terms of a better model fit^59^ and descriptive plots of these indicators across models with different number of classes may show a bend when reaching a well-fitting model.^60,61^ Entropy can take on values between 0 and 1 with higher values showing a higher distinctiveness to other classes.^62^ For the LMR test, *p*-values < .05 imply significant improvement of model fit per each additional class.^57^ As expected, the joint consideration of these fit indices did not provide a clear solution for how many classes fit the observed patterns best. Therefore, we also inspected class diagnostics (i.e. minimal probability of correct, and maximal probability of false class affiliation; number of persons per class) and interpretability of the classes^58^ to identify a parsimonious and balanced model.

For reasons of model performance and consistency, we increased the number of random starts and decided to use the MLF estimator across all models. The MLF estimator provides robust standard errors that are sufficient for parameter interpretation and still allows for comparing models based on fit indices (although not on all, such as the LMR test, thus diverging from the preregistration). Using this estimator made it possible to investigate the latent class structure without dropping some of the social inequality indicators that we wanted to consider.

#### Outcome analyses

In the second step (i.e. outcome analyses), we built on the explorative identification of latent classes and proceeded with the model that was identified as describing the observed patterns best. Using this model, we saved the assignment of people to their classes in a new data file that we used for the outcome analyses. For the outcome analyses, we then assessed the association of latent class membership and nutrition/physical activity app use stage (in separate models each). To do so, we added the respective outcome variable to the identified model as an auxiliary variable (i.e. Bolck-Croon-Hagenaars analysis; Asparouhov and Muthén^63^). This approach avoids class shifts (i.e. keeps the previously identified class membership consistent) and, therefore, allows inspecting differences in nutrition/physical activity app use stage depending on the previously identified latent class membership. Because of the categorical type of the nutrition/physical activity app use stage variable, we tested separate models for each stage (i.e. unengaged non-users, non-users decided to act, non-users decided not to act, acting users and disengaged non-users). Each of these models provided the relative amount of people per class falling into the respective use stage. Consequently, we could descriptively interpret the association of class membership with a specific use stage and identify associated patterns.

## Results

### Sample and descriptive statistics

A comprehensive breakdown of the sample's characteristics can be found in Tables 1 and 2. The overall sample size was *N* = 1974, which was balanced between women (*N* = 979, 49.6%) and men (*N* = 988, 50.1%). Six participants preferred to self-describe their gender (0.3%), and one preferred not to report it (0.1%). Most of the sample lived in a large city, had a citizenship of the respective country, was not part of a minority, held a full-time position and was heterosexual. The median years of education was 13. The median school leaving certification was ‘high school diploma’ in the Italian sample, ‘intermediate secondary school’ in the German and ‘graduation from technical school’ in the Austrian sample. The median vocational certification was ‘apprenticeship’ in the German sample and ‘technical vocational secondary school’ in the Austrian sample. The median household income was €2000/2500. While this value was the same in the Austrian and German samples (i.e. €2500/3000), the median was lower among Italian participants (i.e. €1500/2000).

Table 3 shows the frequencies of physical activity and nutrition app user groups across countries. Overall, unengaged non-users accounted for the majority of both physical activity (*N* = 749, 38.5%) and nutrition users (*N* = 1197, 61.6%).

**Table 3.** Frequency of physical activity and nutrition app user groups for the total sample and by country.

Stages of behavioural adoption	Total		Germany		Austria		Italy	
	*N*	%	*N*	%	*N*	%	*N*	%
App type: Physical activity								
Stage 1: Unengaged non-users	749	38.5	390	40.9	199	41.4	160	31.4
Stage 2: Non-users decided to act	250	12.9	127	13.3	40	8.3	83	16.3
Stage 3: Non-users decided not to act	154	7.9	67	7	31	6.4	56	11
Stage 4: Acting users	539	27.7	258	27	128	26.6	153	30.1
Stage 5: Disengaged non-users	252	13	112	11.7	83	17.3	57	11.2
App type: Nutrition								
Stage 1: unengaged non-users	1197	61.6	585	61.3	321	66.7	291	57.2
Stage 2: Non-users decided to act	222	11.4	109	11.4	33	6.9	80	15.7
Stage 3: Non-users decided not to act	168	8.6	86	9	39	8.1	43	8.4
Stage 4: Acting users	132	6.8	68	7.1	29	6	35	6.9
Stage 5: Disengaged non-users	225	11.6	106	11.1	59	12.3	60	11.8

### Differences in the use of diet and physical activity apps based on social inequality indicators

Tables 4 and 5 show proportions, means, and *p*-values of significant tests across physical activity app and nutrition app adoption stages, respectively.

**Table 4.** Descriptive statistics of correlates of physical activity app adoption.

Social inequality indicator	Stage 1: Unengaged non-users	Stage 2: Non-users decided to act	Stage 3: Non-users decided not to act	Stage 4: Acting users	Stage 5: Disengaged non-users
Gender^a^					
Women *N*	351	128	70	263	152
(res.)	(−1.80)	(−0.48)	(−1.04)	(−0.59)	(−3.67)
Men *N*	393	122	83	276	99
(res.)	−1.8	(−0.48)	−1.04	−0.59	(−3.67)
*p*-value	.072	.628	.301	.595	< .001
Age					
*M*	49.91	41.31	41.91	42.6	40.59
(*SD*)	(15.97)	(14.56)	(13.84)	(15.62)	(15.11)
Years of education					
*M*	13.38	13.42	13.85	14.28	13.93
(*SD*)	(3.00)	(2.97)	(2.82)	(2.86)	(2.83)
Income^b^					
First *N*	200	71	35	103	55
(res.)	(2.30)	(1.83)	(−0.35)	(−3.04)	(−0.83)
*p*-value	.021	.067	.723	.002*	.409
Second *N*	224	72	50	118	55
(res.)	(2.51)	(0.84)	(1.68)	(−2.95)	(−1.88)
*p*-value	.012	.403	.093	.003	.060
Third *N*	260	88	60	258	126
(res.)	(−4.31)	(−1.87)	(−0.48)	(3.98)	(3.19)
*p*-value	.000*	.061	.632	< .000*	.001*
Fourth *N*	65	18	9	59	16
(res.)	(0.10)	(−0.83)	(−1.27)	(−2.30)	(−1.36)
*p*-value	.922	.409	.204	.021	.172
Employment^c^					
Full-time *N*	267	120	77	281	106
(res.)	(−5.72)	(1.44)	(1.62)	(4.6)	(−0.59)
*p*-value	< .000*	.149	.105	< .000*	.557
Part-time *N*	116	38	30	83	49
(res.)	(−0.73)	(−0.48)	(1.13)	(−0.63)	(1.47)
*p*-value	.468	.628	.258	.526	.141
Job-seeking *N*	61	26	14	30	19
(res.)	(0.56)	(1.70)	(0.67)	(−2.20)	(−0.11)
*p*-value	.575	.088	.505	.028	.911
Studying *N*	36	28	12	43	30
(res.)	(−3.75)	(2.25)	(0.06)	(0.32)	(2.71)
*p*-value	<.000*	.024	.951	.748	.007
Home maker *N*	37	11	7	17	14
(res.)	(0.88)	(−0.02)	(0.08)	(−1.69)	(0.94)
*p*-value	.381	.984	.939	.092	.349
Retired *N*	232	27	14	85	34
(res.)	(9.41)	(−3.95)	(−3.57)	(−2.99)	(−2.83)
*p*-value	<.000*	<.000*	<.000*	.003	.005
Country^b^					
Germany *N*	390	127	67	258	112
(res.)	(2.09)	(0.59)	(−1.44)	(−0.66)	(−1.58)
*p*-value	.037	.559	.15	.509	.115
Austria *N*	199	40	31	128	83
(res.)	(1.48)	(−3.43)	(−1.38)	(−0.63)	(3.23)
*p*-value	.140	.000*	.167	.529	.001*
Italy *N*	160	83	56	153	57
(res.)	(−3.83)	(2.70)	(3.00)	(1.37)	(−1.38)
*p*-value	<.000*	0.007	.003*	.171	.168
Discrimination^a^					
Yes	46	31	14	33	19
(res.)	(−1.64)	(3.28)	(0.90)	(−1.32)	(0.16)
No	686	213	135	495	225
(res.)	(1.6)	(−3.28)	(−0.90)	(1.32)	(−0.16)
*p*-value	.101	.001*	.371	.187	.874

*Note: M*: mean; *SD*: standard deviation; YoE: years of education; *N:* number of participants, and the standardized adjusted residuals (in brackets, referred to as “res.”) are displayed.

a Alpha adjusted to *p* = .005 to account for multiple comparisons.

b Alpha adjusted to *p* = .003 to account for multiple comparisons.

c Alpha adjusted to *p* = .002 to account for multiple comparisons.

* sig. *p*-value adjusted based on the number of paired comparisons.^a,b,c^

**Table 5.** Descriptive statistics of correlates of nutrition app adoption.

Social inequality indicator	Stage 1: Unengaged non-users	Stage 2: Non-users decided to act	Stage 3: Non-users decided not to act	Stage 4: Acting users	Stage 5: Disengaged non-users
Gender^a^					
Women *N*	561	107	78	68	150
(res.)	(−3.06)	(−0.50)	(−0.83)	(0.51)	(5.47)
Men *N*	632	115	89	63	74
(res.)	(3.06)	(0.50)	(0.83)	(−0.51)	(−5.47)
*p*-value	.002*	.617	.407	.610	<.000*
Age					
*M*	48.48	41.26	39.06	38.11	38.1
(*SD*)	(15.83)	(15.35)	(13.13)	(14.10)	(14.20)
YoE					
*M*	13.6	13.81	13.91	14.18	14.04
(*SD*)	(2.95)	(2.96)	(2.95)	(2.83)	(2.90)
Employment^c^					
Full-time *N*	489	101	98	76	87
(res.)	(−3.29)	(0.55)	(3.98)	(3.31)	(−1.64)
*p*-value	.001*	0.583	<.000*	.001*	0.1
Part-time *N*	182	41	24	20	49
(res.)	(−1.59)	(0.95)	(−0.72)	(−0.36)	(2.39)
*p*-value	.112	.342	.469	.722	.017
Job-seeking *N*	89	19	15	11	16
(res.)	(−0.59)	(0.50)	(0.62)	(0.28)	(−0.36)
*p*-value	.557	.617	.538	.783	.717
Studying *N*	69	20	13	12	35
(res.)	(−3.99)	(0.80)	(0.04)	(0.64)	(4.73)
*p*-value	<.000*	.424	.970	.523	.000*
Home maker *N*	49	8	7	4	18
(res.)	(−0.90)	(0.63)	(−0.17)	(−0.81)	(2.77)
*p*-value	.370	.527	.865	.420	.006
Retired *N*	319	33	11	9	20
(res.)	(9.02)	(−2.09)	(−4.60)	(−3.96)	(−4.48)
*p*-value	<.000*	.037	<.000*	<.000*	<.000*
Country^b^					
Germany *N*	585	109	86	68	106
(res.)	(−0.23)	(0.01)	(0.57)	(0.58)	(−0.63)
*p*-value	.822	.994	.566	.561	.531
Austria *N*	321	33	39	29	59
(res.)	(2.68)	(−3.62)	(−0.48)	(−0.77)	(0.55)
*p*-value	.007	<.000*	.631	.444	.584
Italy *N*	291	80	43	35	60
(res.)	(−2.38)	(3.55)	(−0.18)	(0.09)	(0.18)
*p*-value	.017	<.000*	.856	.928	.861
Discrimination^a^					
Yes	64	29	19	14	17
(res.)	(−4.36)	(3.43)	(2.14)	(1.45)	(0.20)
No	1108	189	142	116	199
(res.)	(4.36)	(−3.43)	(−2.14)	(−1.45)	(−0.20)
*p*-value	<.000*	.001*	.032	.148	.845
Sexual Orientation^a^					
Heterosexual *N*	98	23	19	27	24
(res.)	(−2.94)	(0.21)	(0.63)	(4.14)	(0.43)
Other *N*	1059	196	146	104	196
(res.)	(2.94)	(−0.21)	(−0.63)	(−4.14)	(−0.43)
*p*-value	.003*	.831	.526	<.000*	.670

*Note: M*: mean; *SD:* standard deviation; YoE: years of education; *N*: number of participants, and the standardized adjusted residuals (in brackets, referred to as “res.”) are displayed.

a Alpha adjusted to *p* = .005 to account for multiple comparisons.

b Alpha adjusted to *p* = .003 to account for multiple comparisons.

c Alpha adjusted to *p* = .002 to account for multiple comparisons.

* sig. *p*-value according to adjustments.^a,b,c^

#### Gender

Overall, both physical activity app use, χ^2^(4, 1937) = 15.08, *p* = .005, *V* = .09, and nutrition app use, χ^2^(4, 1937) = 31.17, *p* < .001, *V* = .18, differed by gender, though the effect sizes were small. Both in the physical activity and nutrition app analysis, women were more frequently disengaged ex-users compared to men. However, men represented most of the unengaged non-users of nutrition apps. No other group comparisons reached statistical significance. Women were more likely than men to have previously used mHealth apps; our hypothesis regarding gender differences was thus confirmed.

#### Age

Since the assumption of homogeneity of variances was violated for both physical activity and nutrition app use stage (Levene's tests – physical activity: *F*(4, 1939) = 4.40, *p* < .05; nutrition: *F*(4, 1939) = 6.42, *p* < .05), Welch's ANOVAs were conducted. Overall age differed according to physical activity app use, *F*(4, 624.76) = 31.83, *p* < .001, η_p_^2^ = 0.06. Pairwise comparisons with Games-Howell correction indicated that unengaged non-users (*M* = 49.91, *SD* = 15.97) were significantly older than non-users who decided to act, non-users decided not to act, active users and disengaged ex-users (*p*s < .001). These results mirror those of nutrition app use, *F*(4, 437.81) = 46.54, *p* < .001, η_p_^2^ = 0.08. Similarly to the physical activity results, unengaged non-users were significantly older (*M* = 48.48, *SD* = 15.83) compared to the other users and non-users’ groups (*p*s < .001). There were no significant differences in age between the other groups in both physical activity and nutrition app use. Older adults were less likely to consider using mHealth apps than younger adults; our hypothesis regarding age differences in nutrition and physical activity app use was thus confirmed. Since the Italian subsample was capped at 50 years of age, we were prompted during peer-review to conduct country-specific analyses. Indeed, there were statistically significant age differences in the Austria and German subsamples in line with the pooled analysis; for Italy, however, there were no statistically significant age differences between groups (fitness apps, using Welch ANOVA: *F*(4, 183.67)* *= 0.90, *p *= .466; nutrition apps: *F*(4, 504)* *= 1.75, *p *= .137).

#### Years of education

Years of education differed significantly by physical activity app use, *F*(4, 1939) = 8.59, *p* < .001, η_p_^2^ = 0.02, but not by nutrition app use, *F*(4, 1939) = 2.12, *p* = .076, η_p_^2^ = 0.00. Active users of physical activity apps reported significantly more years of education (*M* = 14.28, *SD* = 2.85) compared to unengaged non-users (*p* < .001) and non-users who decided not to act (*p* = .001). No significant differences were found between the other groups. Taken together, these results suggest that only for physical activity, adults with a higher level of education are more likely to use apps than adults with a lower level of education, while education is not related to the use of nutrition apps.

#### Net household income

Physical activity app use differed according to income quartiles, χ^2^(12, 1942) = 50.02, *p* < .001, *V* = .09. Participants earning between 5000 and 10,000 Euros (i.e. third quartile) reported a significant smaller rate of unengaged non-users compared to those in the first and second quartile and they accounted for most active users. Specifically, 32.6% of participants in the third quartile group reported themselves as being active users, which was a significantly higher rate than those within the first and second income categories. Similarly, the third quartile earners were also those representing the majority of disengaged ex-users. Thus, physical activity app use is more likely in individuals with higher incomes. Nutrition app use did not vary across income categories χ^2^(12, 1942) = 12.85, *p* = .380, *V* = .05, suggesting that nutrition app use was unrelated to income.

#### Employment

Physical activity app used significantly differed according to employment status, χ^2^(20, 1944) = 127.40, *p* < .001, *V* = .19. Most of the retired participants, homemakers, job seekers, and part-time employees reported themselves being unengaged non-users. Retirees’ percentage of unengaged non-users was significantly higher than that of all the other groups, except for homemakers. Students had a significantly lower percentage of unengaged non-users compared to job seekers, homemakers and retired participants. Full-time workers made up the most active users, although only 33% of all the participants working full-time reported themselves using a physical activity app, significantly differing compared to job seekers, and retired participants.

Nutrition app use varied significantly across employment status, χ^2^(20, 1944) = 126.62, *p* < .001, *V* = .13. Retired participants reported being unengaged non-users more frequently compared to all other employment groups. On the contrary, pensioners were significantly fewer within the non-users decided not to act group compared to participants working full time, seeking employment and students. Full-time workers accounted for the highest proportion of active users. Nonetheless, these participants only represented 8.9% of all participants with a full-time employment, which was significantly higher compared to the retirees. Students reported also a significantly higher proportion of active users compared to the retired group. Likewise, students exhibited a significantly higher rate of disengaged ex-users compared to the full-time workers, the job seekers, and the retired group. Thus, for both physical activity and nutrition apps, retirees are less interested in using them than other adults, while students and full-time employees are more interested and also more likely to use mHealth apps.

#### Rural vs urban residency

Neither physical activity app use, χ^2^(12, 1944) = 16.21, *p* = .182, *V* = .09, nor nutrition app use, χ^2^(12, 1944) = 9.04, *p* = .700, *V* = .04, varied significantly across town size of residence. Rural versus urban residency, thus, is likely unrelated to mHealth app use.

#### Country

Both physical activity, χ^2^(8, 1944) = 40.533, *p* < .001, *V* = .110, and nutrition app use, χ^2^(8, 1944) = 21.99, *p* = .005, *V* = .07, differed significantly across countries. Regarding the former, Italians had a significantly lower proportion of unengaged non-users compared to Austrians and Germans. However, they exhibited a greater rate of non-users who decided not to act, which was significantly higher compared to Austrians and Germans. No country difference was found within the active users’ group, as opposed to the disengaged non-users, where Austrians had a significantly higher rate compared to both Germans and Italians. Concerning nutrition app use, Austrians showed a significantly lower rate of non-users who decided to act than both Germans and Italians, who had the highest. Taken together, Austrian adults may be somewhat less interested in mHealth apps compared to German and Italian adults, although these differences did not translate into actual differences in (former) use.

#### Migration status

Neither physical activity app use, χ^2^ (4, 1944) = 2.14, *p* = .71, *V* = .03, nor nutrition app use, χ^2^(4, 1944) = 4.15, *p* = .392, *V* = .05, differ significantly according to migration background. Migration status, thus, might be of low importance for mHealth app use.

#### Discrimination experiences (proxy for minority status)

Overall, physical activity app use differed significantly by discrimination experience, χ^2^(4, 1897) = 13.01, *p* = .011, *V* = .08. A higher percentage of the participants in the discriminated group reported being non-users who decided to act, as opposed to the non-discriminated group. Moreover, nutrition app user groups also differed in relation to discrimination, χ^2^(4, 1897) = 23.83, *p* < .001, *V* = .11. Despite unengaged non-users being the most common in both discriminated and not discriminated groups, the latter had a higher rate. However, the discriminated group showed higher proportions of non-users who decided to act and non-users decided not to act, compared to the non-discriminated group. Participants self-identifying as a minority were thus more often interested in using mHealth apps, but did not yet put these intentions into action.

#### Sexual orientation

Nutrition app use, but not physical activity up use, χ^2^(4, 1892) = 1.98, *p* = .740, *V* = .03, varied significantly by sexual orientation, χ^2^(4, 1892) = 19.90, *p* < .001, *V* = .10. Most of both heterosexual and non-heterosexual participants reported being unengaged non-users, even though within this user group heterosexual represented the highest proportion. However, heterosexuals were less likely to be active users compared to the non-heterosexual group, indicating that adults who identify as a sexual minority are more interested in and likely to use nutrition apps compared to adults who identify as heterosexual.

### Interplay of social inequality indicators

#### Identification of latent classes

Table 6 shows the fit indices and numbers of participants per latent class for every tested model (i.e. starting with a one-class up to a six-classes solution). Although fit indices did not aggravate strongly for the six-classes solution, we refrained from testing models with more classes, because warning messages for the five- and six-classes solutions already indicated convergence issues (i.e. limitedly interpretability) with the recommendation of reducing the number of classes. In addition, the plot of AIC, BIC and SSA-BIC values showed slight bends for the two-classes, three-classes and four-classes solutions, and afterwards the three curves became clearly flat. From these remaining models (i.e. two-, three- and four-classes solutions), fit indices of the two-classes and four-classes solutions were most appropriate. Both of these models showed the best value in entropy (.81), indicating high distinctiveness between classes, and the smallest probabilities of false class affiliation (Class 2: .09 and Class 3: .10). Although Class 2 had a higher value in minimal correct class affiliation (.91) than Class 4 (.81), we decided to continue the analyses with the four-classes solution, as class distinctiveness was similarly high and this model allowed for a more nuanced pattern. Also, the number of participants per class still indicated a quite parsimonious and balanced model (i.e. no class was extremely small or big), ensuring descriptive interpretability.

**Table 6.** Latent classes of social inequality indicators.

Number of classes	Log-likelihood	FP	AIC	BIC	SSA-BIC	Entropy	Class prob.	Error prob.	*N* C1	*N* C2	*N* C3	*N* C4	*N* C5	*N* C6
1	−24,875.22	18	49,786.44	49,887.03	49,829.84				1974					
2	−24,216.81	34	48,501.62	48,691.61	48,583.59	.81	.91	.09	607	1367				
3	−23,875.75	50	47,851.50	48,130.89	47,972.04	.80	.86	.13	553	494	926			
*4*	***−23,652.04***	***66***	47,436.08	47,804.88	47,595.20	***.81***	***.81***	***.10***	***527***	***495***	***672***	***280***		
5	−23,525.47	82	47,214.94	47,673.15	47,412.63	.80	.82	.11	299	256	482	393	544	
6	−23,411.07	98	47,018.15	47,565.75	47,254.40	.80	.80	.12	300	380	253	464	259	318

*Note*: FP: number of free parameters; AIC: Akaike information criterion; BIC: Bayesian information criterion; SSA-BIC: sample-size-adjusted BIC; Class prob.: minimal probability of correct class affiliation; Error prob.: maximal probability of false class affiliation; *N* C1–6: number of persons per class. Bold and italicized: chosen class solution for further analyses.

Table 7 shows a descriptive and summarized overview of values in social inequality indicators per class for all four classes. Along with the highlighted patterns, we interpret Class 1 (*N* = 527) as mainly including young and diverse citizens (youngest participants, high proportion of migration background and discrimination experiences, mainly not heterosexual), Class 2 (*N* = 495) as mainly including established retirees (oldest participants and mainly retired), Class 3 (*N* = 672) as mainly including economically strong employees (medium age, highest income and education level, and mainly employed), and Class 4 (*N* = 280) as mainly including low-income workers (medium age, lowest income and education level, some studying or being in training) for our further analyses. Interestingly, latent classes were mainly driven by age, employment, income, and education level. Only Class 1 additionally captures differences with regard to migration background, discrimination experiences and sexual orientation. Gender and residency seemed to be equally distributed across classes.

**Table 7.** Descriptive and summarized patterns of social inequality indicators per class.

Social inequality indicator	Class 1	Class 2	Class 3	Class 4
Age	***Youngest* (*average of 26 years*)**	***Oldest*** **(*average of 66 years*)**	Medium (average of 45 years)	Medium (average of 40 years)
Gender	Equally distributed	Equally distributed	Equally distributed	Equally distributed
Migration status and/or discrimination experiences	***High proportion***	Low proportion	Low proportion	Low proportion
Sexual orientation	*** Mainly not heterosexual***	Mainly heterosexual	Mainly heterosexual	Mainly heterosexual
Income	Medium (mainly second quartile)	Medium (mainly second quartile)	***Highest* (*mainly third quartile*)**	***Lowest*** **(*mainly first quartile*)**
Education level	Medium (average of 13.9 years)	Medium (average of 14.2 years)	***Highest* (*average of 14.7 years*)**	***Lowest*** **(*average of 10.3 years*)**
Employment	Mainly part-time employees or people searching for jobs	***Mainly retirees***	Mainly employees (full- or part-time)	Mainly employees (full- or part-time) or people in studies/training
Residency	Rather urban	Rather urban	Rather urban	Rather urban

*Note*: For parsimony, this table shows a descriptive and summarized overview of values in social inequality indicators per class to illustrate the main differences between the classes. The detailed patterns are shown in the shared output files on OSF. Bold and italicized = descriptively noticeable values compared to other classes.

#### Latent class membership and physical activity app use stages

In the next step, we examined whether the four different classes differentially related to specific physical activity app use stages. The results summarized in Table 8 indicate the proportion of participants per class, indicating a specific use stage. In the following, we will focus on the main patterns or outstanding values.

**Table 8.** Four classes based on social inequality indicators as predictors of physical activity app use stages.

Class	Class name	*N*	Stage 1: Unengaged non-users	Stage 2: Non-users decided to act	Stage 3: Non-users decided not to act	Stage 4: Acting users	Stage 5: Disengaged non-users
1	Young and diverse citizens	527	0.25^2,3,4^	0.16^2,(3)^	0.08^2^	0.32^2,4^	0.18^2,3,(4)^
2	Established retirees	495	***0.59^1,3,4^***	0.08^1,3,4^	0.04^1,3,4^	0.22^1,3^	0.08^1,3,4^
3	Economically strong employees	672	0.33^1,2,4^	0.13^(1),2^	0.10^2^	0.32^2,4^	0.13^1,2^
4	Low-income workers	280	***0.43^1,2,3^***	0.16^2^	0.10^2^	0.18^1,3^	0.13^(1),2^

*Note*: The table shows the proportion of participants per class, indicating a specific use stage, adding up to ∼1 (due to rounding) per row. Superscripts indicate significant differences at *p* < .05 between the respective classes (e.g. superscript 2 = difference to Class 2). Superscripts in brackets indicate a marginally significant difference at *p* < .10 between the respective classes. *Bold and italicized* *=* *relatively clear patterns (i.e. high proportion of participants within one class (>40%), indicating a specific use stage).*

Class 1, including young and diverse citizens, had the lowest proportion of unengaged non-users (Stage 1, 25%) compared to all other classes, while Class 2, including established retirees, had the highest proportion of them (59%). Moreover, established retirees had the lowest proportion of non-users who decided to act (Stage 2, 8%), of non-users who decided not to act (Stage 3, 4%), as well as of disengaged non-users (Stage 5, 8%). For Class 3, including economically strong employees, as well as for Class 4, including low-income workers, most of the participants were also unengaged non-users (Stage 1, 33% and 43%, respectively). A critical difference between these two classes, however, was that the economically strong employees had a significantly higher proportion of acting users (Stage 4, 32%) than the class including low-income workers (18%). Relatedly, the young and diverse citizens had a similarly high proportion of acting users (32%) as the economically strong employees, while the proportion of acting users in established retirees (22%) was not higher than in the low-income workers. Thus, we observed that acting users were mostly included in the Classes with economically strong employees (Class 3) and in the class including young and diverse citizens (Class 1). For the young and diverse citizens, acting users even reflected the highest proportion of a specific app use stage.

#### Latent class membership and nutrition app use stages

Using the same approach, we then examined whether the four different classes differentially related to specific nutrition app use stages. The results summarized in Table 9 again indicate the proportion of participants per class, indicating a specific use stage. In the following, we will focus on the main patterns or outstanding values.

**Table 9.** Four classes based on social inequality indicators as predictors of nutrition app use stages.

Class	Class name	*N*	Stage 1: Unengaged non-users	Stage 2: Non-users decided to act	Stage 3: Non-users decided not to act	Stage 4: Acting users	Stage 5: Disengaged non-users
1	Young and diverse citizens	527	***0.45^2,3,4^***	0.15^2,3^	0.11^2^	0.11^2,3,4^	0.18^2,3,4^
2	Established retirees	495	***0.81^1,3,4^***	0.09^1,(4)^	0.02^1,3,4^	0.03^1,3,(4)^	0.05^1,3,4^
3	Economically strong employees	672	***0.61^1,2^***	0.10^1^	0.11^2^	0.07^1,2^	0.11^1,2^
4	Low-income workers	280	***0.62^1,2^***	0.13^(2)^	0.08^2^	0.05^1,(2)^	0.11^1,2^

*Note*: The table shows the proportion of participants per class, indicating a specific use stage, adding up to ∼1 (due to rounding) per row. Superscripts indicate significant differences at *p* < .05 between the respective classes (e.g. superscript 2 = difference to Class 2). Superscripts in brackets indicate a marginally significant difference at *p* < .10 between the respective classes. *Bold and italicized* *=* *relatively clear patterns (i.e. high proportion of participants within one class (>40%), indicating a specific use stage).*

For all four classes, unengaged non-users (Stage 1) reflected the highest proportion of a specific app use stage. Looking at this pattern more nuancedly, however, showed that Class 2, including the established retirees, had the highest proportion of unengaged non-users (81%) compared to all other classes, while Class 1, including the young and diverse citizens, had the relatively lowest proportion of unengaged non-users, although they were still at 45%. This latter class also reflected the highest relative proportion of acting users (Stage 4, still only 11%) as well as of disengaged non-users (Stage 5, 18%). Class 3, including economically strong employees, and Class 4, including low-income workers, did not significantly differ from each other in app use stages, but only with respect to Classes 1 and 2. For both economically strong employees and low-income workers, almost two-thirds were unengaged non-users (61% and 62%, respectively), and the fewest participants in these classes were acting users (7% and 5%, respectively).

## Discussion

Using data from nationally representative samples for the adult populations in Austria, Germany and Italy, this survey indicates that adoption rates of nutrition and physical activity apps are rather low across countries and that app use is associated with certain social inequality indicators. In line with prior research that mainly stemmed from the United States, younger individuals with a higher level of education and higher income are more likely to use nutrition and physical activity apps. This analysis thus provides further evidence for the existence of a digital health divide, with more deprived populations using mHealth apps less than less deprived populations.^24,25^ Mobile health promotion programmes may therefore risk widening existing health inequalities.

### Characterisation of mHealth app (non-)users based on a conjoint analysis of social inequality indicators

Extending prior research, which typically focused on a small set of social inequality indicators and investigated their associations with mHealth app use separately (see e.g. König et al.^26^ for a summary), this analysis tested the conjoint effects of several social inequality indicators on mHealth app use to identify independent core predictors. Two groups of participants could be identified who were most likely to currently use nutrition and fitness apps. One group was characterised by young age. Indeed, age differences in mHealth app (non-)use are consistently reported in the literature, especially when real-life adoption of mHealth interventions is studied.^24^ These age differences are also in line with age differences in using technology in general^64^ and point towards the potential role of experience with and affinity to technology.^65^ Although results from randomised-controlled trials indicate that older adults can benefit from digital interventions just as much as younger adults,^25^ these settings are highly specific. First, there may be a selection bias, with participants being more likely to enrol in a study if motivated to use technology and perceive it as beneficial.^66^ Second, participants likely received more training and guidance within the study than if they would download an app by themselves. This points towards the crucial role of healthcare professionals as potential gatekeepers^67^ who need to be appropriately trained in digital health for this task.^68^ At the same time, another recent German survey also highlights that digital health literacy^69,70^ is especially important for health technology use in older adults,^71^ which underscores training needs in laypeople to address this age gap.

The second group that used mHealth apps more frequently was mainly characterised by the highest income and education levels. Again, this mirrors prior research indicating that higher socio-economic position (SEP) is associated with increased digital intervention uptake and effectiveness.^26,72^ SEP is also associated with improved health outcomes in general; it could thus be assumed that these associations simply mirror general inequalities in healthcare.^73^ However, SEP is also associated with access to digital technology.^22^ Introducing digital technology into healthcare, therefore, might further increase these existing inequalities. Again, the results highlight that increasing digitisation of the healthcare system has to be implemented with caution and should ideally be accompanied by additional (regulatory) means, such as improving access to relevant technology and promoting digital literacy.^21^

### Extension by rarely studied social inequality indicators: Sexual orientation and migration status

The present analysis is also among few that included a broad range of social inequality indicators based on the PROGRESS-Plus framework,^23^ some of which have previously been mostly neglected.^25,26^ Indeed, regarding sexual orientation, the present analysis indicates that individuals who identify as non-heterosexual may be more likely to use nutrition apps, which could be due to expected or actually experienced discrimination in the healthcare context,^74^ or increased familiarity with other digital health promotion and care tools, which may spill over into the domain of nutrition.^75^ Yet, this finding diverges from one prior study that found no association between sexual orientation and nutrition app uptake during the COVID-19 pandemic.^76^ However, the effect size in the present study was small, and the result, therefore, may have only reached statistical significance because of the large sample size. Further research, both on mHealth app use among populations with diverse sexual orientations and reasons for use, such as discrimination experience in the healthcare system,^74^ should be conducted.

Similarly, the present analysis is one of few to consider migration status. Contrary to prior research, migration status was unrelated to mHealth app use in the present study. Part of the reasons for the diverging findings could be different definitions of migration status (e.g. based on native language vs. immigration; cf. Ernsting et al.^15^ and Pevnick et al.^77^). Since the present study was conducted only in the countries’ respective official language, individuals who do not speak this language well were systematically excluded from participating. To shed further light on the relationships between migration and mHealth app use, studies should be conducted in the respondents’ native languages to improve reach.

### Gender effects

Importantly, our study highlights the relevance of using diverse methodological approaches to study the digital health divide comprehensively. For example, we found that gender indeed plays a role in mHealth app adoption (i.e. more female disengaged non-users in physical activity and nutrition apps, more male unengaged non-users in nutrition apps) when tested independently. Women using mobile interventions more frequently is a common finding in the literature,^26^ potentially because women are more interested in health topics such as healthy diets than men.^78^ However, gender differences were not detected in the conjoint analyses. This deviation may be due to our representative sample, in which the proportion of men and women was fairly balanced and probably did not vary greatly across the other variables (e.g. age, education level and income). In turn, gender might not have been recognised as a separation criterion in the latent class analyses, especially not in comparison to other variables that showed higher variation. Therefore, it is crucial to remind that our study aimed to identify *both* critical predictors of mHealth app adoption per se as well as critical subgroups in the population that predict mHealth app adoption. While the identification of predictor variables alone may overestimate their relevance since interrelations with other relevant variables are neglected, the identification of subgroups may omit potentially relevant single predictors.

### Strengths and limitations

The present analysis has several strengths, including the relatively large and nationally representative samples from three European countries. By recruiting nationally representative samples, we provide data on population subgroups that are typically underrepresented in (digital health) research, such as men, individuals of lower SEP, and older adults.^79,80^ We also conducted an extensive assessment of socio-demographic characteristics that may be associated with mHealth app adoption. These characteristics were studied both individually and conjointly to identify the most prominent predictors of using nutrition and physical activity apps. Given that results largely align with findings from other geographical areas, most notably the United States,^24,25^ results likely generalise across countries, at least within the Global North.^21^

Yet, several limitations of the study have to be acknowledged. Most notably, while our findings regarding the class characterised by young age thus tie in well with existing literature, it is important to consider that the age distribution across classes might have been biased due to the slightly restricted age range in the Italian sample. Given the overall large sample size and fit to existing literature, we see it as unlikely that this restriction affected our core results considerably. Yet, it is important to note that there were no age differences between app user groups when testing for age differences in the Italian sample only, which indicates that at least for adults aged 50 years and younger, differences in use rates are negligible. Efforts to increase digital intervention use, thus, may indeed need to focus on older adults aged significantly above 50 years. Similarly, for historical reasons, all three countries do not record races or ethnicities. The assessment of minority status thus had to be relatively superficial, yet followed recommendations by a diversity assessment consortium to also ensure comparability with other future studies.^54^

Although the sample is large and the study is thus adequately powered to detect small effects, no formal a priori power calculation had been conducted. The study was observational and did not investigate underlying mechanisms of the identified associations; while it seems plausible that less modifiable characteristics, such as socio-demographic characteristics, influence more modifiable characteristics such as mHealth app use, this causality could not be formally tested with this approach.

Research on the underlying (psychological) mechanisms of the digital health divide is generally scarce and should be prioritised to identify potential means for addressing this issue.^25,81,82^ Indeed, mHealth app adoption may be affected by app functions and content.^83^ The variety of apps available nowadays might allow for a better fit between subgroups of the population (e.g. based on age, and indicated by the difference between young and diverse citizens vs. established retirees) and specific design (e.g. more functions vs. easier usage). Furthermore, participants were recruited via a panel provider focusing on conducting online studies. Participants thus may have at least basic digital literacy, and the study, therefore, may slightly overestimate the proportion of mHealth app users in the general population. Furthermore, while certain indicators were necessarily evaluated as dichotomous variables (e.g. migration status and experiences of discrimination), others were *analysed* dichotomously (e.g. gender and sexual orientation). This methodological choice was driven by the limited statistical power of the subcategories within these indicators. This was a consequence of the inability to recruit a truly representative diverse sample with respect to gender identities, due to the strictly binary nature of Italian and Austrian national statistics, and given the fact that the majority of the populations indeed identified using binary gender categories. We were therefore unable to draw conclusions regarding individuals who identify as another or no gender. Future research should aim to specifically target and oversample these subgroups to explore a broader spectrum of gender identities and sexual orientations.

Finally, the present analysis included a broad range of applications, that is, both commercial and medical, as well as apps primarily used for monitoring versus apps primarily used for coaching. Furthermore, we did not specify a minimum use frequency. Although prior work suggests that factors driving inequalities at the uptake versus engagement stages,^26^ more fine-grained analyses are needed to disentangle conjoint effects of inequality indicators for the decisions to download or install an app versus the frequency or intensity of engagement, as well as whether engagement was truly meaningful (cf. Perski et al.^84^).

### Implications for policy and practice

Given current national policy efforts that are undertaken in many countries, including Germany and Austria, actions need to be taken that explicitly address the digital health divide. For example, the Digital Healthcare Act implemented in Germany in 2019 allows doctors to prescribe digital therapeutics, which will then be covered by health insurance.^85,86^ Prescription and reimbursement are contingent upon proof of efficacy; yet, there is no explicit statement regarding proof of efficacy across population subgroups, or even requirements to involve relevant patient groups in the development process.^21^ Similarly, healthcare professionals are often not appropriately trained in using digital technology; persisting stereotypes may thus further exacerbate inequalities. Guidelines and requirements for educational programmes thus urgently require adjustments, for example, by including modules on social and digital determinants of health in the curriculum and the inclusion of relevant assessments into practitioners’ workflows.^68^

## Conclusions

In principle, digital health technology may contribute to health for all, yet low uptake rates that are unevenly distributed in the population currently strongly limit their public health benefit. Especially older adults with lower income and education levels may currently not benefit from available mobile tools for promoting healthier diets and physical activity. Tailored approaches and policy action are urgently needed to address this gap and to avoid widening existing health inequalities through the inevitable digitization of health promotion and care.
